# Real‐world health utility scores and toxicities to tyrosine kinase inhibitors in epidermal growth factor receptor mutated advanced non‐small cell lung cancer

**DOI:** 10.1002/cam4.2603

**Published:** 2019-10-24

**Authors:** Shirley Xue Jiang, Ryan N. Walton, Katrina Hueniken, Justine Baek, Alexandra McCartney, Catherine Labbé, Elliot Smith, Sze Wah Samuel Chan, RuiQi Chen, Catherine Brown, Devalben Patel, Mindy Liang, Lawson Eng, Adrian Sacher, Penelope Bradbury, Natasha B. Leighl, Frances A. Shepherd, Wei Xu, Geoffrey Liu, Manjusha Hurry, Grainne M. O'Kane

**Affiliations:** ^1^ Division of Medical Oncology and Hematology Princess Margaret Cancer Centre University of Toronto Toronto ON Canada; ^2^ Faculty of Medicine University of Toronto Toronto ON Canada; ^3^ AstraZeneca Canada Mississauga ON Canada; ^4^ Insitut Universitaire de Cardiologie et de Pneumologie de Québec Université Laval Québec QC Canada; ^5^ Biostatistics Princess Margaret Cancer Centre and Dalla Lana School of Public Health University of Toronto Toronto ON Canada; ^6^ Epidemiology Dalla Lana School of Public Health University of Toronto Toronto ON Canada

**Keywords:** EGFR mutation, health economics, health utility scores, lung cancer, real‐world

## Abstract

**Background:**

As the treatment landscape in patients with non‐small cell lung cancer (NSCLC) harboring mutations in the epidermal growth factor receptor (*EGFR*m) continues to evolve, real‐world health utility scores (HUS) become increasingly important for economic analyses.

**Methods:**

In an observational cohort study, questionnaires were completed in *EGFR*m NSCLC outpatients, to include demographics, EQ‐5D‐based HUS and patient‐reported toxicity and symptoms. Clinical and radiologic characteristics together with outcomes were extracted from chart review. The impact of health states, treatment type, toxicities, and clinical variables on HUS were evaluated.

**Results:**

Between 2014 and 2018, a total of 260 patients completed 994 encounters. Across treatment groups, patients with disease progression had lower HUS compared to controlled disease (0.771 vs 0.803; *P* = .01). Patients predominantly received gefitinib as the first‐line EGFR tyrosine kinase inhibitor (TKI) (n = 157, mean‐HUS = 0.798), whereas osimertinib (n = 62, mean‐HUS = 0.806) and chemotherapy (n = 38, mean‐HUS = 0.721) were more likely used in subsequent treatment lines. In longitudinal analysis, TKIs retained high HUS (>0.78) compared to chemotherapy (HUS < 0.74). There were no differences between the frequency or severity of toxicity scores in patients receiving gefitinib compared to osimertinib; however, TKI therapy resulted in fewer toxicities than chemotherapy (*P* < .05), with the exception of worse diarrhea and skin rash (*P* < .001). Severity in toxicities inversely correlated with HUS (*P* < .001). Clinico‐demographic factors significantly affecting HUS included age, Eastern Cooperative Oncology Group Performance Score (ECOG PS), disease state, treatment group, and metastatic burden.

**Conclusions:**

In a real‐world *EGFR*m population, patients treated with gefitinib or osimertinib had similar HUS and toxicities, scores which were superior to chemotherapy. Health utility scores inversely correlated with patient‐reported toxicity scores. In the era of targeted therapies, future economic analyses should incorporate real‐world HUS.

## Graphical abstract

Real‐world health utility scores (HUS) in epidermal growth factor receptor mutations (EGFRm) non‐small cell lung cancer outpatients were similar between those treated by first‐ and second‐line tyrosine kinase inhibitors, which were superior to chemotherapy. Treatment‐associated toxicities corresponded to HUS of treatment groups.

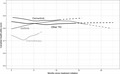

## 1 INTRODUCTION

Mutations in the epidermal growth factor receptor gene (*EGFR*) are the most common and actionable molecular aberrations in non‐small cell lung cancer (NSCLC).^1^, ^2^, ^3^ Epidermal growth factor receptor tyrosine kinase inhibitors (EGFR‐TKIs) are effective in the treatment of advanced lung cancers harbouring EGFR mutations (*EGFR*m),^4^, ^5^, ^6^, ^7^ but remain expensive especially given continuous dosing for the duration that a patient benefits from these TKIs.^8^, ^9^

Efficacy of these EGFR‐TKIs is not in question. First generation (gefitinib, erlotinib) and second generation (afatinib, dacomitinib) EGFR‐TKIs have demonstrated improved progression‐free survival (PFS), objective response rates, and tolerability when compared to first‐line platinum doublet chemotherapy.^4^, ^5^, ^6^, ^7^ However, acquired resistance to EGFR‐TKIs develops over time. The most common mechanism of resistance leading to failure of first‐ and second‐generation TKIs is a secondary mutation in the *EGFR* gene, Thr790Met (T790M), found in up to 60% of patients.^10^, ^11^ Osimertinib, a third‐generation TKI, is effective as second‐line treatment in patients with a confirmed T790M mutation, directly targeting this acquired resistance.^12^, ^13^ Recently, osimertinib was found effective as first‐line treatment to improve PFS compared to first generation TKIs (18.9 months vs 10.2 months, hazard ratio 0.46; 95% CI 0.37‐0.57; *P* < .001).^14^, ^15^

These rapid advances in EGFR targeted therapies have led to improved clinical outcomes;^16^, ^17^, ^18^ however, the associated increased cost renders cost‐effectiveness studies critical, particularly in public healthcare systems.^19^, ^20^, ^21^ Cost‐effectiveness studies rely on quality‐adjusted life years, which are based on health utility scores (HUS).^22^ However, HUS data are sparse in the *EGFR*m NSCLC population for several reasons. Although HUS and health‐related quality of life (HRQoL) data are often collected in clinical trials, trial patients are not representative of the corresponding real‐world patient population. In particular, there is a lack of real‐world longitudinal data, which is important as HUS have been shown to fluctuate with disease course.^23^ Furthermore, most existing real‐world observational studies report HUS broadly for NSCLC,^24^, ^25^, ^26^ failing to account for mutational status and disease state, which have also been demonstrated to affect quality of life.^27^

While TKIs are associated with fewer adverse effects compared to chemotherapy, the longer time on therapy may result in chronicity and decreases in health utility.^28^ When the health states of a general advanced NSCLC population were examined, commonly observed symptoms such as cough, dyspnea, and pain corresponded with a mean utility loss of 0.069.^26^ Within an *EGFR*m‐specific population, poor appetite and fatigue have been associated with lower HUS.^27^ Furthermore, recent studies have demonstrated that treatment‐related adverse effects correlate with different quality of life across TKI agents; however, these data are limited to gefitinib, erlotinib, and afatinib.^29^, ^30^ While there have been new advances in EGFR*m* treatments and it has been shown that different treatments have implications on HUS, there remains limited data on the impact of treatment and toxicities in an *EGFR*m population.

Health utility scores are commonly generated by EQ‐5D, a standardized measure that can be applied in various health and treatment states. The EQ‐5D instrument has been validated in a general population as a generic preference‐weight measure.^31^ In contrast, *EGFR*m‐NSCLC populations are enriched for never‐smokers, females, and Asians,^32^ differing substantially from the composition of the general, healthy North American population. Even within lung cancer patients, *EGFR*m patients significantly depart from the typical heavy smoking stereotype.^33^ Describing real‐world HUS in *EGFR*m patients under various health states while evaluating the impact of clinico‐demographic factors and toxicities on HUS is necessary to generate data that can be used confidently in cost‐effectiveness analyses of this subset of NSCLC patients.

## 2 MATERIALS AND METHODS

### 2.1 Patients

This cohort study was approved by the University Health Network Institutional Research Ethics Board (Toronto, Canada). Participants were outpatients at Princess Margaret Cancer Centre who had advanced, histologically confirmed *EGFR*m‐NSCLC from November 2014 to July 2018. Participants were all able to provide informed consent and could enroll at any point during their disease course. Longitudinal data were obtained through follow‐up surveys collected at regular outpatient visits scheduled by the physician.

### 2.2 Questionnaires

The baseline questionnaire included multiple components. A demographic questionnaire collected data surrounding age, sex, ethnicity, and social factors. An EQ‐5D questionnaire was comprised of five dimensions (mobility, self‐care, usual activity, pain or discomfort, anxiety, or depression), whereby the patient would evaluate their health state on a scale of problem levels ranging from “no problems” to “extreme problems” for each dimension. A single digit score is generated for each dimension and all five dimension scores can be combined using a predetermined formula for a final number between 0 and 1 describing the patient's health state. The visual analogue scale allows patients to self‐rate their health from “the worst healthy you can imagine” (0) to “the best health you can imagine” (100). The baseline questionnaire also evaluated treatment‐related symptoms using a questionnaire based on the Patient‐Reported Outcomes version of the Common Terminology Criteria for Adverse Events (PRO‐CTCAE). In addition to the most relevant PRO‐CTCAE items to EGFR‐TKIs as identified by expert review (such as diarrhea, fatigue, nausea/vomiting), we included a specific skin toxicity section for inclusion of specific areas affected by and details of non‐acneform rashes. Frequency and severity of toxicities were described using a scoring system similar to PRO‐CTCAE reporting for each treatment group, whereby a numerical score was assigned to represent the magnitude of severity and frequency of the toxicity item (Table S1). In this scoring, 1 represents absence of any toxicity and 5 represents the most severe or frequent toxicity; with the exception of hair loss and visual disorders, which utilized scores ranging from 1 to 3; and skin rash, which utilized a score between 1 and 4. Follow‐up surveys included the EQ‐5D questionnaire and the toxicity questions. Additional patient‐reported outcome measures for all subjects were extracted from the Edmonton Symptom Assessment System‐Distress Assessment and Response Tool (ESAS‐DART),^34^ which is a validated electronic self‐assessment symptom screening tool routinely administered to cancer patients at Princess Margaret Cancer Centre at all clinic visits. ESAS‐DART provides a score on an 11‐point scale from 0 (no symptoms) to 10 (most severe symptom) for 10 key symptoms: pain, tiredness, drowsiness, nausea, appetite, shortness of breath, depression, anxiety, well‐being, and functional status.

### 2.3 Chart abstraction and variable classification

Clinical data were extracted from medical records, including diagnostic tests, diagnosis date, and stage at diagnosis. Extracted treatment information included all surgical, radiation, and systemic treatments. Regular imaging at Princess Margaret Cancer Centre documented whether patients had radiologic evidence of treatment response, stability, or progression.

Health states were classified into stable or progressive disease. Stable disease was defined by not having any recorded evidence of disease progression radiologically, pathologically or clinically. Progressive disease was defined by reported radiologic, pathologic, or clinical progression documented on the patient's medical record. Progressive disease was further subdivided into patients demonstrating disease progression while being maintained on the same TKI (ie treatment beyond progression), or demonstrating disease progression where the current TKI was stopped or switched at progression (ie no treatment beyond progression). Toxicity data were categorized as mild, moderate, or severe based on the PRO‐CTCAE data, as classified in Table S1.

### 2.4 Statistical analysis

Descriptive summary statistics are reported for individual patients, stratified by systemic treatment type (referred to as “treatment group”). Pairwise comparisons evaluated associations between various treatment group definitions and HUS, using *t* tests. Trends in HUS over time since treatment initiation were plotted using locally weighted polynomial least squares regression (LOESS), stratified by treatment group.

Differences between treatment groups for each toxicity were explored using ANOVA (mean severity) and Kruskal‐Wallis tests (proportion of patients reporting moderate to severe toxicity). Pairwise comparisons were then conducted to further explore significant associations between individual toxicities and treatment group and a Bonferroni correction was used to account for multiple comparisons. Boxplots were created to describe associations between each toxicity/ESAS symptom and HUS. Spearman correlational coefficients were generated to describe associations between individual toxicities/symptoms and HUS. Boxplots were also used to visualize trends in HUS when grouped by most severe reported toxicity score or ESAS symptom per clinic visit, stratified by treatment group.

This study collected data at each patient's clinic visit (referred to throughout this paper as “encounters”); therefore, one patient could contribute multiple HUS at different time points and across varying lines of treatment. To account for multiple observations per patient in univariable and multivariable analyses, a single mean HUS was calculated per patient within each “health state,” defined as all encounters per patient on a single treatment, within a single disease status (stable or progressing on treatment). For example, patient who contributed HUS both before and after progressing on a single treatment would contribute two “health states” to the analysis on that given treatment: one mean HUS of all scores generated in stable disease, and a second for all scores after disease progression occurred on that treatment.

To examine whether EQ‐5D‐generated HUS reflected patient's health state in *EGFR*m‐NSCLC patients, ANOVA was conducted to evaluate unadjusted associations between HUS by health state and key clinical factors. Associations between treatment group and HUS per health state were adjusted for potential confounders using multivariable linear regression. A preliminary regression model was fit using clinic‐demographic variables significantly associated with HUS, excluding treatment group, followed by backwards selection. Finally, treatment group was added into the result.

Statistical significance was defined as *P* < .05, with the exception of pairwise comparisons for which the Bonferroni adjustment was applied and statistical significance was *P* < .00125. All analyses were conducted using R, version 3.4.3.

## 3 RESULTS

### 3.1 Baseline characteristics

Our study included 260 *EGFRm*‐NSCLC outpatients, who provided HUS in 994 clinical encounters; after controlling for each individual patient providing the same health state over multiple visits, we defined 488 individual health states as stable or progressing. The median number of encounters completed by each participant was 3 (range 1‐15); participation rate was 87% of all patients approached. Baseline characteristics and treatment received at first encounter for each patient at study entry are reported in Table 1. Throughout the study, 157 patients received gefitinib (149 in first line, 8 subsequent line), 62 received osimertinib (3 first line, 59 subsequent line), 59 received another TKI (19 first line, 40 subsequent line), and 38 were treated with chemotherapy (4 in first line, 34 in subsequent line). Eighty‐three patients contributed HUS when not on treatment and 5 contributed HUS on immunotherapy (n = 4) or other treatment (n = 1) throughout the study duration, wherein 1 patient contributed encounters to both no treatment and other treatment.

**Table 1:** Baseline patient characteristics by treatment at first encounter

Characteristic: N (%)	Gefitinib	Osimertinib	Other TKI	Chemo‐therapy	None/other^a^	*P*‐value
Number of patients on treatment at first encounter (study entry)	129	14	33	18	66	
Age at diagnosis of stage IV disease: mean [SD]	66[13]	59 [11]^b^	62 [13]	57 [11]^b^	64 [14]	**.03**
Stage IV at diagnosis: N (%)	77 (60)	13 (93)^b^	18 (56)	15 (83)	36 (55)	**.02**
Gender						
Female	86 (67)	10 (71)^b^	23 (70)	14 (78)^b^	38 (58)	.50
Male	43 (33)	4 (29)	10 (30)	4 (22)	28 (42)	
Ethnicity						
Asian	80 (62)	6 (43)	12 (38)^b^	6 (33)^b^	36 (55)	**.03**
Other	13 (10)	3 (21)	9 (28)	2 (11)	8 (12)	
Smoking status at stage IV diagnosis						
Ever smoker	42 (33)	2 (14)	8 (25)	8 (47)	20 (31)	.36
Never smoker	87 (67)	12 (86)	24 (75)	9 (53)	45 (69)	
ECOG performance status at stage IV diagnosis						
0	43 (33)	4 (29)	13 (39)	6 (33)	18 (27)	.65
1	75 (58)	10 (71)	20 (61)	12 (67)	44 (67)	
2+	11 (9)	0 (0)	0 (0)	0 (0)	4 (6)	
Lines of treatment: first line, N (%)	123 (95)	0 (0)^b^	14 (42)^b^	2 (11)^b^	56 (85)^b^	**<.001**
Number of metastatic sites involved:^c^ mean [SD]	2.1 [1.2]	2.4 [1.0]	2.0 [1.1]	2.9 [1.2]^b^	1.9 [1.1]	**.02**
Bone metastasis^c^: N (%)	62 (48)	10 (71)	15 (46)	12 (67)	25 (38)	.08
Brain metastasis^c^: N (%)	50 (39)	8 (57)	6 (18)^b^	11 (61)	23 (35)	**.02**
Pleural effusion^c^: N (%)	49 (38)	4 (29)	15 (46)	8 (44)	23 (35)	.76
Liver metastasis^c^: N (%)	17 (13)	4 (29)	5 (15)	8 (44)^b^	8 (12)	**.01**
Nodal metastasis^c^: N (%)	75 (58)	7 (50)	23 (70)	13 (72)	42 (64)	.52
Adrenal metastasis^c^: N (%)	11 (9)	1 (7)	3 (9)	0 (0)	7 (11)	.77
Total number of patients on treatment at any encounter/total encounters in study)^d^	157/419	62/195	59/169	38/87	87/124	

a Of 66 total patients, 65 were not on treatment and one was on immunotherapy.

b *P* < .05 on pairwise comparison with reference of gefitinib, using Fisher's exact (categorical variables) or *t* tests (continuous variables).

c At first encounter.

d The total number of patients on each treatment sums to more than n = 260 because some patients contributed data to the study on multiple treatments.

Significant *P*‐values (< 0.05) were bolded.

Significant differences in demographic and clinical characteristics between treatment groups were found. Patients undergoing gefitinib treatment were older (mean 65.8 years) while patients receiving osimertinib (mean 59.0 years) and chemotherapy (mean 57.2 years) were younger (*P* = .03). Line of therapy was significantly different between treatment groups as gefitinib was typically used in the first‐line setting; osimertinib, chemotherapy, and other TKIs were generally given at failure of gefitinib (*P* < .001). More metastatic sites were associated with chemotherapy (mean 2.89) when compared to gefitinib (mean 2.05) (*P* = .01) and patients treated with chemotherapy also were more likely to have acquired liver metastasis (*P* = .01). In the osimertinib treatment group, significantly more patients had advanced stage disease (defined as stage IV) at time of first diagnosis (92.9%), compared to patients treated with gefitinib, who were more likely to present at an earlier stage and later develop metastatic disease (59.7% diagnosed at stage IV, 40.3% early‐stage; *P* = .02).

### 3.2 Disease state and HUS

Mean HUS of all encounters according to disease states and treatment group are shown in Table 2. As expected, across all treatment groups, stable disease (295 individual health states) was associated with higher mean HUS compared to disease progression (193 individual health states) (0.803 vs 0.771; *P* = .05). Gefitinib, osimertinib, and chemotherapy also had consistently numerically higher mean HUS during disease stability, when compared with disease progression. In an exploratory analysis of HUS in patients treated beyond progression, no significant differences in HUS were found between health states of patients who continued their treatment beyond disease progression (n = 58) compared to health states when treatment was stopped or switched (n = 58). The clinical strategy of continuing a TKI beyond progression in selected patients was thus not associated with lower HUS, when compared to stopping the current treatment at progression (0.775 vs 0.739, respectively; *P* = .26).

**Table 2:** Mean health utility scores by health state, according to disease state and treatment

Treatment group	All disease states;	Stable disease^a^	Progressing disease	*P*‐value comparing Stable vs progressing disease	Progressing disease^b^		*P*‐value comparing Treatment beyond vs not after progression
	mHUS (N)	mHUS (N)	mHUS (N)		Continuing treatment beyond progression	No treatment beyond progression	
All health states	0.783 (n = 488)	0.795 (n = 295)	0.764 (n = 193)	**.05**			
All health states while on treatment (chemo or TKI)	0.790 (n = 405)	0.803 (n = 242)	0.771 (n = 163)	.06	0.775 (n = 58)	0 0.739 (n = 58)	.26

Drug	mHUS (N)	mHUS (N)	*P*‐value comparing across drugs, stable disease	mHUS (N)	mHUS (N)	mHUS (N)
Gefitinib	0.798 (n = 209)	0.810 (n = 121)	Reference	0.780 (n = 88)	0.769 (n = 40)	0.749 (n = 29)
Osimertinib	0.806 (n = 81)	0.815 (n = 54)	.86	0.786 (n = 27)	0.817 (n = 7)	0.808 (n = 5)
Other TKI	0.796 (n = 69)	0.795 (n = 42)	.61	0.798 (n = 27)	0.764 (n = 10)	0.783 (n = 10)
Chemotherapy	0.721 (n = 46)	0.756 (n = 25)	**.04**	0.678 (n = 21)	0.826 (n = 1)	0.661 (n = 14)

Abbreviations: mHUS, mean health utility score; N, number of encounters; TKI, tyrosine kinase inhibitor.

a The definition of stable disease refers to any non‐progressive disease, and includes patients with true stable disease and those responding to disease. Where there were comparisons involving small numbers of patients, statistical analyses are not performed and no *P*‐values are therefore presented.

b Treatment beyond progression analysis was conducted in a subset of patients for whom clinical strategy was clear from chart review. All encounters recorded where patients were treated beyond progression were further classified as continuing treatment beyond progression and no treatment beyond progression (ie stopping or switching treatment).

Significant *P*‐values (< 0.05) were bolded.

### 3.3 Treatment and HUS

Considering only patients with stable disease in Table 2, all treatment groups were compared with gefitinib, as it was the most commonly used treatment. Comparatively, osimertinib was associated with similar mean HUS (mean HUS of 0.810 gefitinib vs 0.815 osimertinib; *P* = .78). Those treated with chemotherapy had lower mean HUS (mean HUS of 0.756; *P* = .04). Finally, other TKI treatment (primarily, afatinib, and erlotinib) had a mean HUS in between chemotherapy and gefitinib/osimertinib (mean HUS of 0.795). To account for different treatment lines, a univariable analysis was conducted between second‐line treatment groups that also demonstrated similar mean HUS between osimertinib, gefitinib, and other TKIs, which were superior to chemotherapy.

**Figure 1:**
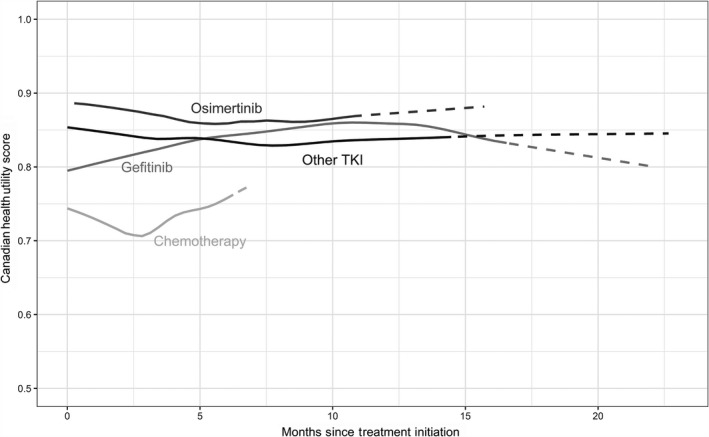
Mean health utility scores over time according to treatment for patients with stable or responding disease. Locally weighted polynomial least squares regression (LOESS) was used to generate plots. The vertical axis represents the mean health utility scores using Canadian reference weights when patients were stable on treatment. The horizontal axis represented time in months since treatment initiation. Dashed lines on the left side represent the period when <15% of patients have begun contributing health utility scores (HUS) data on treatment; on the right, the dashed sections represent HUS where <15% of patients continue to contribute data. Thus, both left and right sides represent censoring beyond the point at which <15% of the sample is contributing HUS data. Dashed sections of the curve indicate areas where substantial selection bias could occur. Other TKI (tyrosine kinase inhibitors) represents a heterogeneous group of therapies that included afatinib, erlotinib, and TKIs undergoing clinical trial

The LOESS regression curve in Figure 1 represents HUS in patients with stable disease who were treated with gefitinib, osimertinib, other TKIs, and chemotherapy over time, since their treatment was initiated. After excluding the first few months of therapy, which may represent time to stabilization of disease, disease symptoms, initial toxicity symptoms, and HRQoL, there was consistency in the longitudinal data; TKI treatments were similar, retaining durable HUS > 0.75 with osimertinib and gefitinib retaining HUS > 0.80 up to approximately 1 year on treatment. Chemotherapy was consistently associated with the lowest HUS, substantially lower than the cluster formed by the TKI therapies; as the majority of chemotherapy patients progressed within a few months of therapy, longitudinal data beyond a few months was very limited.

### 3.4 Clinical variables and HUS

The role of clinico‐demographic factors affecting HUS is outlined in Table 3. In univariable analysis, higher mean HUS was associated with both lower ECOG performance status (ECOG PS) at the time of stage IV diagnosis (*P* < .001), and being in a stable disease state (*P* = .01). Higher mean HUS was also associated with the absence of bone (*P* = .004), liver (*P* = .02) and pleural (*P* = .04) metastases and a lower number of metastatic sites (*P* = .02). Notably, mean HUS did not decrease with brain metastasis. Mean HUS were not significantly associated with line of treatment (*P* = .56) but HUS did vary by current systemic treatment (*P* = .01).

**Table 3:** Clinico‐demographic factors affecting HUS from all encounters

Covariate	Category	N = 488^a^	Univariable analysis		Multivariable analysis^b^	
			Mean HUS by health state (95% CI)	*P*‐value	Change in HUS, regression β (95% CI)	*P*‐value
Age at diagnosis of stage IV^c^	65+	304	0.79 (0.77‐0.81)	.17	−0.002 (−0.003, 0)	**.007**
	Under 65	184	0.77 (0.74‐0.79)			
Sex	Male	162	0.79 (0.76‐0.81)	.63		
	Female	326	0.78 (0.76‐0.79)			
ECOG PS at stage IV	0	168	0.83 (0.80‐0.86)	**<.001**	Reference	
	1	297	0.76 (0.74‐0.78)		−0.051 (−0.08, −0.02)	**.001**
	2+	23	0.67 (0.62‐0.72)		−0.13 (−0.20, −0.06)	**<.001**
Disease state	Stable	295	0.80 (0.78‐0.81)	**.01**	Reference	**0.006**
	Progressing	193	0.76 (0.73‐0.78)		−0.042 (−0.07, −0.01)	
Line of treatment	1	278	0.79 (0.77‐0.81)	.56		
	2	118	0.77 (0.74‐0.80)			
	3+	92	0.77 (0.74‐0.81)			
Treatment at encounter^b^	Gefitinib	209	0.79 (0.77‐0.81)	**.01**	Reference	
	Osimertinib	81	0.80 (0.77‐0.84)		0.006 (−0.04, 0.05)	.78
	Chemotherapy	46	0.73 (0.68‐0.77)		−0.062 (−0.12, −0.01)	**.02**
	Other TKI	69	0.80 (0.77‐0.83)		0.0006 (−0.04, 0.04)	.98
	None/other	83	0.74 (0.70‐0.78)		−0.048 (−0.09, −0.01)	**.01**
Number of metastatic sites^c^	0	24	0.79 (0.71‐0.88)	**.02**	−0.014 (−0.03, −0.002)	**.03**
	1	116	0.81 (0.79‐0.84)			
	2	160	0.79 (0.77‐0.82)			
	3	104	0.76 (0.72‐0.79)			
	4	65	0.74 (0.69‐0.78)			
	5+	19	0.73 (0.65‐0.80)			
Brain metastasis	Yes	218	0.78 (0.76‐0.80)	.73		
	No	270	0.78 (0.76‐0.80)			
Bone metastasis	Yes	245	0.76 (0.74‐0.78)	**.004**		
	No	243	0.80 (0.78‐0.82)			
Liver metastasis	Yes	89	0.74 (0.70‐0.78)	**.02**		
	No	399	0.79 (0.77‐0.80)			
Pleural effusion or metastasis	Yes	200	0.76 (0.74‐0.79)	**.04**		
	No	288	0.79 (0.77‐0.81)			
Nodal metastasis	Yes	307	0.77 (0.75‐0.79)	.15		
	No	181	0.79 (0.77‐0.82)			

a Number of health states per line of therapy per person to account for multiple observations per patient.

b The multivariable model presented was created by entering all variables significant at *P* = .05, with the exception of treatment at encounter. After backwards selection, the resultant model was created that then forced treatment at encounter back into the multivariable model; it is this final multivariable model that is being presented.

c This variable was entered into the multivariable analysis as continuous. It has been reported in categories for the univariable analysis to show distributions of HUS.

Significant *P*‐values (< 0.05) were bolded.

In multivariable analysis, lower HUS was significantly associated with more advanced age at stage IV diagnosis (*P* = .007), a higher ECOG score at first diagnosis (*P* < .01), having disease progression at the time of assessment (*P* = .006), and having an increasing number of metastatic sites (*P* = .03; see Table 3). Bone, liver, and pleural metastases were not found to contribute to the regression model, thus no metastatic sites were included in the final MVA. After adjusting for clinically relevant variables and using gefitinib as the reference, HUS was similar across all different types of TKI therapy while HUS was inferior in patients who were treated with chemotherapy (*P* = .02) or not on treatment (*P* = .01).

### 3.5 Frequency and severity of treatment‐related toxicities

Table 4 summarizes the frequency and severity of 10 common toxicities (scored according to Supplementary Table 1) reported at any encounter, separated by treatment group. After Bonferroni adjustment, significance was defined as *P* < .00125. Compared to gefitinib, osimertinib had lower mean severity of skin rash (*P* = .001); all other treatment‐related toxicities were similar between osimertinib and gefitinib treatment groups. Patients who were untreated (n = 83) or on another treatment (n = 5), had significantly less severe diarrhea and skin rash compared to gefitinib, which are established side effects of TKIs. When comparing gefitinib and chemotherapy, the first line TKI was associated with less severe decreased appetite, nausea, vomiting, and fatigue; however, chemotherapy had superior profile for skin rash and diarrhea (*P* < .0013). A similar trend was seen when examining the frequency of all toxicities. There was no significant difference in toxicity frequency between gefitinib, osimertinib, and other TKIs (*P* > .0012); however, chemotherapy was associated with a higher frequency of reported constipation, decreased appetite, nausea, vomiting, and fatigue (*P* < .0012).

**Table 4:** Toxicities by treatment

	Gefitinib			Osimertinib			Other TKI			Chemotherapy			None/other			*P*‐value^c^	*P*‐value^d^
	Any (%)	Grade 3‐5 (%)	Mean Grade [SE]	Any (%)	Grade 3‐5 (%)	Mean grade [SE]	Any (%)	Grade 3‐5 (%)	Mean grade [SE]	Any (%)	Grade 3‐5 (%)	Mean grade [SE]	Any (%)	Grade 3‐5 (%)	Mean grade [SE]		
Number of encounters	N = 243			N = 127			N = 97			N = 52			N = 64			Any reported toxicities by treatment (%)	Mean severity of toxicities by treatment
Number of patients	N = 113			N = 56			N = 44			N = 27			N = 45				
Diarrhea	72	40	2.27 [0.07]	71	40	2.23 [0.09]	78	47	2.46 [0.11]	50	23	1.75 [0.12]^a^	50	22	1.77 [0.12]^a^	<.001	<.001
Constipation	54	26	1.88 [0.07]	42	12	1.54 [0.10]	44	23	1.82 [0.14]	82^b^	25	2.25 [0.16]	63	45	2.26 [0.16]	.002	.002
Decreased appetite	46	19	1.71 [0.06]	58	29	1.99 [0.09]	61	29	2.05 [0.11]	73^b^	46	2.40 [0.16]^a^	64	30	2.09 [0.14]	<.001	<.001
Nausea	34	12	1.49 [0.05]	30	10	1.45 [0.07]	44	9	1.58 [0.09]	81^b^	44	2.40 [0.14]^a^	42	17	1.72 [0.13]	<.001	<.001
Vomiting	19	4	1.23 [0.03]	25	5	1.30 [0.05]	22	5	1.28 [0.06]	54^b^	35	1.94 [0.14]^a^	33	13	1.48 [0.10]	<.001	<.001
Fatigue	78	37	2.24 [0.06]	80	37	2.30 [0.09]	89	51	2.61 [0.10]	98^b^	73	2.96 [0.11]^a^	89	44	2.58 [0.13]	<.001	<.001
Neuropathy	33	11	1.45 [0.06]	46	13	1.59 [0.11]	55	22	1.82 [0.12]	54	31	1.92 [0.16]	40	23	1.67 [0.14]	.02	.01
Skin rash	75	46	2.26 [0.08]	45	26	1.71 [0.14]^a^	72	40	2.19 [0.13]	47	22	1.69 [0.14]^a^	30^a^	19	1.51 [0.13]^a^	<.001	<.001
Visual disorders	42	11	1.53 [0.04]	64	19	1.83 [0.07]	48	14	1.62 [0.08]	53	16	1.69 [0.10]	42	8	1.50 [0.08]	.001	.002
Hair loss	37	15	1.52 [0.06]	21	8	1.29 [0.10]	36	17	1.53 [0.11]	69	28	1.97 [0.13]	30	9	1.40 [0.10]	<.001	<.001

a *P* < .00125 on pairwise comparison with gefitinib as reference, using *t* tests to compare mean scores.

b *P* < .00125 on pairwise comparison with gefitinib as reference, using Mann‐Whitney *U* tests to compare raw scores.

c *P*‐values computed across all treatment groups using Kruskal‐Wallis method.

d *P*‐values computed across all treatment groups using ANOVA (mean severity).

Severity of treatment toxicity from TKIs, including gefitinib, and osimertinib, were compared to chemotherapy in Table S2. As expected, chemotherapy was associated with significantly or numerically higher severity of constipation, decreased appetite, nausea, vomiting, fatigue, neuropathy, and hair loss (*P* < .05). However, chemotherapy was again associated with lower severity of diarrhea (*P* = .001) and skin rash (*P* = .008), which was more common with TKI use.

### 3.6 Toxicities and HUS

**Figure 2:**
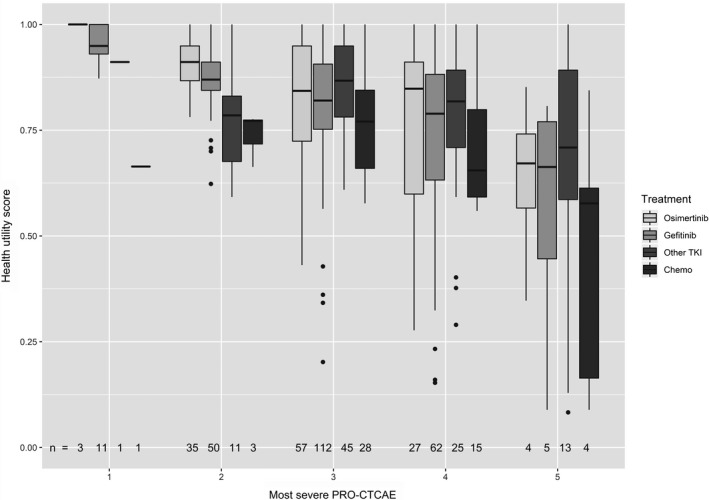
Association between health utility scores and toxicity severity, by treatment. Boxplots of health utility score across each treatment and the most‐severe toxicity score (where 1 represents absence of any toxicities assessed, while 5 represents very severe toxicity. Health utility scores were generated using the Canadian reference weights. TKI, tyrosine kinase inhibitors

The box plots in Figure 2 illustrate the relationship between the most severe reported toxicity among all 10 toxicities and their corresponding HUS, according to treatment group. There was an overall trend where increasing severity of the most‐severe toxicity corresponded to poorer HUS. When examining HUS by treatment group, chemotherapy was consistently associated with the lowest median HUS, when compared to all of the TKI treatment groups, across each level of the most severe reported toxicity.

**Figure 3:**
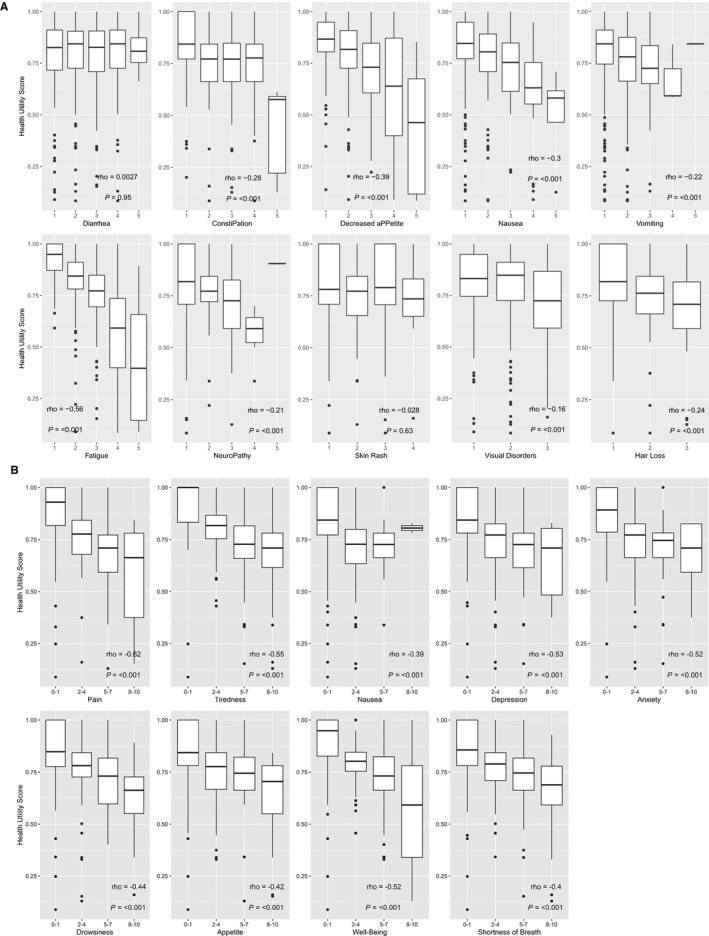
A, Association between health utility scores (HUS) and toxicities. All toxicity scores utilized a scale from 1 through 5 except for hair loss and visual disorders, which utilized a scale between 1 and 3, and skin rash which utilized a scale between 1 and 4. Rho and *P*‐values were generated from Spearman correlational analyses. B, Association between HUS and Edmonton Symptom Assessment System (ESAS) toxicities. ESAS (Edmonton Symptom Assessment System) symptom scores utilized a scale from 0 through 10. Rho and *P*‐values were generated from Spearman correlational analyses

Figure 3A demonstrates the relationship between HUS and severity of individual symptoms and toxicities, across all treatment groups. Increasing toxicity severity scores correlated with decreasing HUS for each of the individual symptoms (*ρ* = −0.16 to −0.56), except for diarrhea (*ρ* = 0.003) and skin rash (*ρ* = −0.03). To further explore the relationships between HUS and toxicities specifically associated with TKI treatment (ie diarrhea, skin rash), treatment groups were separated into common TKIs (combining gefitinib and osimertinib) and chemotherapy, using Spearman correlation to describe the trends in HUS among patients on each treatment in Figure S1. This figure suggests that all toxicities except skin rash in TKI‐treated patients had some degree of association with HUS.

The ESAS tool was completed by 160 (62%) of all outpatients at any clinical encounter during their treatment. Figure 3B shows that higher overall severity score collected by the ESAS tool was associated with lower HUS (*ρ* = −0.39 to −0.62; all *P* < .001), with the rho values trending higher than those seen with the reported toxicity scores.

## 4 DISCUSSION

In this real‐world study of patients with *EGFR*m NSCLC, HUS and patient‐reported toxicities were similar across various TKIs, which were superior to patients on chemotherapy or no treatment. In longitudinal analysis, chemotherapy was associated with the lowest HUS, while both osimertinib and gefitinib retained high HUS over time with mean HUS values above 0.80, as long as disease remained nonprogressing. It is important to note that patients treated with chemotherapy may intrinsically have a lower HUS as these patients may be further in their disease course. However, despite also being used in a subsequent line‐setting, osimertinib was associated with high HUS and in this real world setting, a mean HUS of 0.815 for stable disease and 0.786 at progression, are comparable to the AURA2 clinical trial data that reported mean HUS of 0.812 and 0.751, respectively.^35^ For all TKIs, progression was associated with a mean fall in HUS of approximately ≤ 0.03, less than a previously reported minimally clinically important difference in EQ‐5D utility of 0.06‐0.08.^36^ There are three possible explanations for this small drop in mean HUS. First, some patients may have oligoprogression, whereby tumor heterogeneity affords many patients continued benefit from the current TKI treatment despite progression of some clones.^37^ Second, the high HUS derived from prior disease stability during TKI therapy may provide a reserve to allow patients to better tolerate disease progression. Third, *EGFR*m disease progression may include a subset that tends to be slower, leading to milder disease progression symptoms than other forms of NSCLC. It is likely that all three explanations contributed to this relatively small fall in HUS at the time of disease progression. Nonetheless, these *EGFR*m‐specific explanations argue that this subpopulation of patients needs to be treated and analyzed separately from other forms of lung cancer.

We found that our real‐world patient‐reported symptoms (ESAS) and toxicity grading (similar to PRO‐CTCAE) generated data closely resembling reports from clinical trials. From our toxicity scores, we found that higher frequency and severity of diarrhea and skin rash were the only toxicities worsened with TKI treatment compared to chemotherapy or no treatment. This expands upon a meta‐analysis of RCTs examining gefitinib, erlotinib, afatinib, and chemotherapy that found rash and diarrhea to be the most frequent TKI‐specific toxicities.^38^ In contrast, patients treated with chemotherapy were more likely to develop nausea, vomiting, fatigue and loss of appetite, which is consistent with chemotherapy treatment in wild‐type NSCLC patients.^39^ The cumulatively higher number and severity of toxicities associated with chemotherapy treatment also explains why there was a greater negative impact of chemotherapy on HUS than with TKI therapy, when analysis were stratified by the single worst symptom/toxicity (Figure 2). Finally, regarding the new agent osimertinib, the FLAURA study^14^ reported less grade 3 toxicities of osimertinib compared to standard TKI therapy (34% vs 45%) when used in a first‐line setting. In our real‐world study, osimertinib was used primarily in a second‐line setting, suggesting greater burden of disease in patients given progression on first‐line treatment; however, osimertinib did not have worse toxicities scores compared to other TKI treatments and had less severe skin rash compared to gefitinib (*P* < .0013). Skin rash however did not impact HUS underscoring the need to carefully analyse the impact of toxicities in clinical studies. In addition, when comparing toxicities and symptoms with HUS, we identified mild‐to‐moderate inverse correlations between HUS and the majority of toxicity scores (*ρ* = 0.16 to −0.56), as well as ESAS (*ρ* = −0.39 to −0.62). This further supports the similarities in HUS incurred across TKI treatments when considering toxicity as an important partial driver of HUS.^29^ However, the absence of very strong correlations suggests that more than just one single symptom or driver impacts HUS.

Furthermore, we identified important differences in common symptom and toxicity questionnaires which may impact HUS: for example, ESAS scores of anxiety, depression, and well‐being (*ρ* > 0.5) are not captured by the patient‐reported toxicity questionnaire used, but are captured to some degree within EQ5D, supporting slightly stronger correlations between ESAS and HUS. Another explanation for stronger correlations between ESAS symptoms and HUS may be that the inclusion of these generic symptoms applies to all patients as opposed to specific toxicities similar to PRO‐CTCAE. Thus, a deeper understanding of patient‐reported outcome data, especially how it impacts HUS, is still required and should be captured within clinical trials.

We verified the correlation between HUS and relevant clinico‐demographic factors such as age, ECOG performance score, metastatic burden, disease state, and treatment group in the real world.^26^, ^27^ Particularly, in this *EGFR*m enriched population, we did not find that the presence of brain metastases had a significant impact on HUS, despite brain involvement and their associated treatments being commonly regarded as a significant cause of morbidity.^40^ This supports a recent article, which demonstrated an effect on HUS by mutational subtype, prior radiation and disease control rather than presence of brain metastases.^41^ While bone metastases appeared to significantly affect HUS on UVA, they were not found to contribute overall to HUS in our MVA. This challenges the potential impact of bone metastases on HRQoL, such as previous report of bone as a particularly challenging site for pain control and treatment,^42^ further the need for real‐world HUS. Given the correlation between HUS to clinically relevant factors and symptoms reported in *EGFR*m patients, our study demonstrates that the HUS generated by EQ‐5D are clinically appropriate to use for economic analyses in this subpopulation of advanced lung cancer and should be included in cost‐effectiveness studies.

There are several limitations to our study. As our study population consisted of cancer outpatients, there may be a recruitment bias towards fitter (ie nonhospital) patients who were able to attend clinic, with sicker patients declining to participate. Furthermore, as one patient was able to contribute multiple encounters, those who felt better and attended more clinics could contribute more data points. Our study assessed patients within a single specialized cancer care center, which may limit the generalizability of our findings to geographic regions that have very different resources and clinical practices. Similarly, given the numerous treatment options offered at our center, the patient population may include more individuals with higher disease burden and on subsequent lines of treatment compared to other centers. This is reflected in the baseline demographic characteristics of our study population. To mitigate this, we limited our analysis to focus on gefitinib, osimertinib, and chemotherapy as major treatment groups. This heterogeneity in the patient population studied is also a limitation when comparing toxicity and symptoms across line of therapy. However, we conducted a sensitivity analysis of specific line to line treatment, which demonstrated similar results as reported, and a MVA examining the effect of clinico‐demographic factors on toxicity score did not show significant association between treatment line and toxicity severity. Furthermore the similar profiles between osimertinib and gefitinib despite the use of osimertinib predominantly in second line would support the more favourable side effect profile of osimertinib from the FLAURA study,^14^ given its use in treatment naive patients.

## 5 CONCLUSION

In a real‐world Canadian *EGFR*m‐NSCLC population, we found that first‐line gefitinib and third‐generation osimertinib had similar mean HUS and patient‐reported toxicity and symptom scores. All TKI treatments had higher mean HUS compared to chemotherapy which was durable over time and was minimally disturbed by disease progression. Patient‐reported toxicities and symptoms correlated with lower HUS across all treatment groups, demonstrating the importance of tempering toxicities to improve HUS. Our analyses also support the application of EQ‐5D in the real world setting as a robust means for generating HUS in the *EGFR*m population. As *EGFR*‐targeted treatment continues to expand, more real‐world studies are needed to assess corresponding toxicities and impact on HUS to facilitate reliable cost‐effectiveness analyses for new and existing treatments.

## Data Availability

All datasets used and analyzed during the current study are available from the corresponding author on reasonable request.
