# Supplementary material for: Real‐world health utility scores and toxicities to tyrosine kinase inhibitors in epidermal growth factor receptor mutated advanced non‐small cell lung cancer
Source: Cancer Med. 2019 Oct 24;8(18):7542–55. doi: 10.1002/cam4.2603 (PMC6912023; doi:10.1002/cam4.2603)

Association of PRO-CTCAE Toxicities with HUS for Main TKIs (Gefitinib/Osimertinib) vs. Chemotherapy

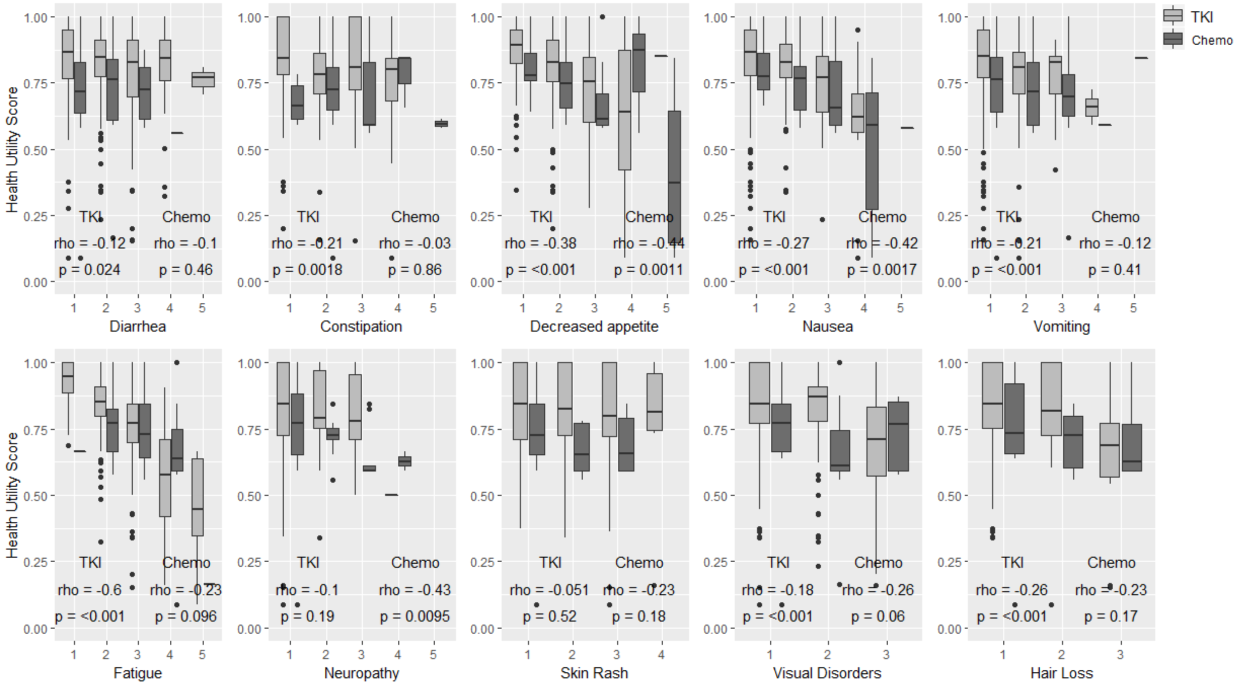

Supplement: Supplementary file 1 [file CAM4-8-7542-s001.pdf]
