# Supplementary material for: Real‐world health utility scores and toxicities to tyrosine kinase inhibitors in epidermal growth factor receptor mutated advanced non‐small cell lung cancer
Source: Cancer Med. 2019 Oct 24;8(18):7542–55. doi: 10.1002/cam4.2603 (PMC6912023; doi:10.1002/cam4.2603)
Supplement: Supplementary file 2 [file CAM4-8-7542-s002.docx]

**Supplementary Table 1: Individual Symptom/Toxicity Score Definitions**

| **Symptom** | **Severity Score** | | | | |
| --- | --- | --- | --- | --- | --- |
|  | **1** | **2** | **3** | **4** | **5** |
| **Diarrhea** | Never | Rarely | Occasionally | Frequently | Almost Constantly |
| **Vomiting** | Never | Rarely | Occasionally | Frequently | Almost Constantly |
| **Constipation** | Never | Mild | Moderate | Severe | Very Severe |
| **Decreased Appetite** | Never | Mild | Moderate | Severe | Very Severe |
| **Nausea** | Never | Mild | Moderate | Severe | Very Severe |
| **Fatigue** | Never | Mild | Moderate | Severe | Very Severe |
| **Neuropathy** | Never | Mild | Moderate | Severe | Very Severe |
| **Skin Rash** | No | Yes, asymptomatic | Yes, with symptoms of pruritus or pain, but tolerable | Yes, with symptoms, interfering with daily life |  |
| **Visual Disorders** | No | Yes | Yes, interfering with usual activities |  | |
| **Hair Loss** | No | Yes, < 50% of the hair | Yes, > 50% of the hair |  |  |

All toxicity measures were assigned a score from 1 through 5 based on frequency and severity, where 1 represents absence of any toxicity and 5 represents most severe or frequent toxicity; exceptions are hairloss and visual disorders, which utilized scores ranging from 1 to 3, and skin rash, which utilized scores between 1 to 4.

**Supplementary Table 2: Toxicities of Common TKIs (Gefitinib and Osimertinib) vs. Chemotherapy Treatment**

| **Toxicities** | **Common TKIs Combined (Gefitinib and Osimertinib)**  N= 365 encounters | | **Chemotherapy**  N=51 encounters | | **P-value** |
| --- | --- | --- | --- | --- | --- |
|  | Mean Grade | SE | Mean Grade | SE |  |
| **Diarrhea** | 2.25 | 0.05 | 1.75 | 0.12 | **0.001** |
| **Constipation** | 1.81 | 0.06 | 2.25 | 0.16 | **0.007** |
| **Decreased appetite** | 1.81 | 0.05 | 2.40 | 0.16 | **<0.001** |
| **Nausea** | 1.48 | 0.04 | 2.40 | 0.14 | **<0.001** |
| **Vomiting** | 1.25 | 0.03 | 1.94 | 0.14 | **<0.001** |
| **Fatigue** | 2.26 | 0.05 | 2.96 | 0.11 | **<0.001** |
| **Neuropathy** | 1.48 | 0.05 | 1.92 | 0.16 | **0.002** |
| **Skin Rash** | 2.14 | 0.07 | 1.69 | 0.14 | **0.008** |
| **Visual Disorders** | 1.63 | 0.04 | 1.69 | 0.10 | 0.62 |
| **Hair Loss** | 1.46 | 0.06 | 1.97 | 0.13 | **<0.001** |
| **Most Severe Toxicity Score**^†^ | 2.98 | 0.04 | 3.38 | 0.11 | **0.002** |
| ^†^**Indicates the most severe reported toxicity reported per encounter.** | | | | | |

Supplementary Table 3. Mean HUS by Second-Line Treatments

| Treatment | Gefitinib | Osimertinib | Other TKI | Chemotherapy |
| --- | --- | --- | --- | --- |
| N^†^ | 8 | 34 | 22 | 18 |
| Mean HUS^‡^ | 0.801 | 0.802 | 0.684 | 0.788 |
| P-value | Ref | 0.98 | 0.81 | **0.03** |

^†^Sample size comprised of patients on second line treatment in any treatment group.

^‡^Mean HUS during stable disease encounters for each patient per line of therapy, to account for multiple observations per patient.
